# Improving Emotion Perception in Children with Autism Spectrum Disorder with Computer-Based Training and Hearing Amplification

**DOI:** 10.3390/brainsci11040469

**Published:** 2021-04-08

**Authors:** Joan H. Leung, Suzanne C. Purdy, Paul M. Corballis

**Affiliations:** School of Psychology, The University of Auckland, Auckland 1023, New Zealand; sc.purdy@auckland.ac.nz (S.C.P.); p.corballis@auckland.ac.nz (P.M.C.)

**Keywords:** Autism Spectrum Disorder, speech prosody, auditory processing, hearing amplification, training intervention, cortical auditory evoked potentials

## Abstract

Individuals with Autism Spectrum Disorder (ASD) experience challenges with social communication, often involving emotional elements of language. This may stem from underlying auditory processing difficulties, especially when incoming speech is nuanced or complex. This study explored the effects of auditory training on social perception abilities of children with ASD. The training combined use of a remote-microphone hearing system and computerized emotion perception training. At baseline, children with ASD had poorer social communication scores and delayed mismatch negativity (MMN) compared to typically developing children. Behavioral results, measured pre- and post-intervention, revealed increased social perception scores in children with ASD to the extent that they outperformed their typically developing peers post-intervention. Electrophysiology results revealed changes in neural responses to emotional speech stimuli. Post-intervention, mismatch responses of children with ASD more closely resembled their neurotypical peers, with shorter MMN latencies, a significantly heightened P2 wave, and greater differentiation of emotional stimuli, consistent with their improved behavioral results. This study sets the foundation for further investigation into connections between auditory processing difficulties and social perception and communication for individuals with ASD, and provides a promising indication that combining amplified hearing and computer-based targeted social perception training using emotional speech stimuli may have neuro-rehabilitative benefits.

## 1. Introduction

Autism Spectrum Disorder (ASD) is a lifelong, pervasive, neurodevelopmental condition characterized by restrictive, repetitive patterns of behavior and deficits in social language and communication [1]. People with ASD often experience difficulty understanding irony, conflicting emotions, jealousy, social blunders, and others’ intentions [2,3]. Accordingly, a number of researchers have suggested that the core language impairments in ASD reflect problems with language pragmatics [4,5,6,7,8]. Pragmatics, here, refers to the social, emotional, and communicative elements of language, which include nonverbal communicative cues such as prosody—the minimal distinctions in spoken language that convey the speaker’s emotion and intent [9,10].

The perception of prosody is thought to depend on a number of acoustic parameters such as variations in fundamental frequency (pitch), intensity, timbre, and timing [11,12,13,14]. Electrophysiological studies indicate that the human auditory system engages immediately with prosodic cues while processing spoken language [15], and that specific cortical regions respond to variation in affective prosody (i.e., emotion) [16].

Because perception of prosody relies on the accurate processing of subtle variations in acoustic parameters, the perception of the subtle nuances in social or emotional speech can be impaired by difficulties with auditory pattern recognition, auditory discrimination, sound localization, and temporal processing [17]. It is likely that at least a portion of the difficulties in prosodic processing experienced by people with ASD can be attributed to impaired central auditory processing of spoken language. Over the past two decades, a number of researchers have documented atypical auditory processing in individuals with ASD [18,19,20,21]. While some studies show that children with ASD have superior pitch discrimination and categorization in music processing [22,23], they do not show the same advantage in responses to speech [24].

Auditory processing challenges become more salient in individuals with ASD when the input is more complex, such as with tasks involving sentences and intonation [25], speech recognition in the presence of background noise [26,27], auditory filtering [28], competing speech streams [29,30], and when prosody-based cues are processed simultaneously with semantic information [31,32]. These challenges are more disruptive in children with ASD than they are in adults [21].

Electrophysiological studies of individuals with ASD have largely measured cortical auditory evoked potentials (CAEPs), and have mainly focused on ascertaining whether individuals with ASD possess the auditory processing abilities to detect prosodic changes, for example in phonemic intensity, pitch, and duration [33,34,35], and emotional undertones in the speaking voice [36,37,38]. Studies have demonstrated that delayed responses (longer latencies) across various CAEP components are correlated with impaired vocal affect recognition [39,40]. Studies investigating pre-attentive processing of vocal emotion [41,42,43] have typically calculated a mismatch response (MMR) from CAEPs recorded in an oddball experimental paradigm. The MMR is derived by subtracting evoked potentials elicited in response to a numerously repeated “standard” stimulus, from evoked potentials elicited in response to a less frequently and randomly presented “deviant” stimulus [44].

The foregoing suggests that training that targets central auditory processing—particularly for the acoustic discrimination abilities involved in prosodic processing—could ameliorate problems in social communication in people with ASD, perhaps addressing secondary problems such as academic underachievement, inattention, hypersensitivity, hyperactivity, and oppositional behavior [45], as well as noise sensitivity and anxiety [46]. Training studies with children with ASD [47,48,49] have demonstrated measurable post-training improvements, both in terms of improved behavioral performance [47,48] and in faster neural responses [49].

Several research groups have pursued interventions using sound amplification technology—remote-microphone hearing systems (RMHSs)—with the aim of managing the auditory functioning of children with ASD and other language and learning disorders [50,51,52,53,54,55,56]. These studies have reported benefits to academic performance in noisy classroom environments, reduced listening difficulties, improved speech recognition, and improved spatial and temporal processing of auditory stimuli [50,51,52,53,54,56], as well as decreased cortisol levels, indicative of reduced stress [55].

In this study, we trial an intervention in a group of children with high-functioning ASD in which we combine and RMHSs with a computerized training program in emotion perception. There is growing interest in this approach of enhancing traditional auditory training tasks with hearing amplification devices, with innovative studies from the University of North Texas reporting behavioral [53] and neuro-electrophysiological improvements [56].

We seek to expand on the emerging view that the difficulties people with ASD experience in perceiving and understanding subtle nuances in speech may, in part, stem from underlying auditory processing difficulties. We present behavioral and electrophysiological results, measured pre- and post-intervention, from the ASD group and a control group of typically developing (TD) peers who were only assessed at one time point. We aimed to address the following questions:

1. Do social perception abilities differ between the TD and ASD groups?
2. Do these differences change after the training intervention?
3. Do neural responses to changes in emotional undertones in speech differ between the TD and ASD groups?
4. Do neural responses change after the training intervention?

## 2. Materials and Methods

### 2.1. Ethical Approval

All participants gave their written informed consent for inclusion before they participated in this study. This study was approved by the University of Auckland Human Participants Ethics Committee (reference number 9657).

### 2.2. Participants

We recruited two groups of children—an ASD group and a TD control group. The ASD group consisted of 12 children with existing diagnoses of ASD previously made by an individual or multidisciplinary team of health care practitioners. As part of the eligibility criteria for this study, the children with ASD were also evaluated with a rating scale of autism symptoms to re-confirm their diagnosis. There were 9 males and 3 females (*M* = 9.91 years, *SD* = 2.23 years, *Range* = 7–13 years). Two children in this group had comorbid diagnoses of dyslexia (they were twins), and one child had ADHD. Of the 12 children with ASD, two did not assent to the evoked potential recording sessions, therefore CAEP results are only available for 10 of the 12 children with ASD.

The TD group consisted of 14 children with no current or previous diagnosis of any clinical disorders, as confirmed by their parents/caregivers. There were 4 males and 10 females (*M* = 9.43 years, *SD* = 1.87 years, *Range* = 7–12 years).

Participants in this study were all born in New Zealand, and would have been exposed to verbal cues, and prosodic intonation and expression specific to New Zealand English. All participants were enrolled in mainstream school. Two children with ASD required teacher aides to assist them in a group setting (not one-on-one assistance), but otherwise, there was a strong indication that participants with ASD were managing well in class with peers of the same age. A medium to high level of SES was also assumed for the participants in this study, since the public schools (where students enroll by residential zones) had the resources to cater for students with learning difficulties.

### 2.3. Study Design

We used a prospective longitudinal study design to evaluate intervention outcomes for children with ASD. We assessed participants with ASD four times, twice before and twice after an intervention period of three weeks. Session 1 served as a baseline assessment. Evaluations of autism symptoms and communication behaviors were completed. The children completed a hearing screen, a behavioral social perception assessment, and we recorded their baseline CAEPs.

Two weeks later, Session 2 involved the re-assessment of the behavioral social perception task only and served as a second baseline reference point. During this session, we fitted and verified RMHSs for each child with ASD in preparation for the 3 week intervention period.

Session 2 was followed by a week-long familiarization period, during which the ASD group were introduced to using RMHS and briefed about the upcoming intervention. They also received various aids to support this familiarization, including social stories, step-by-step instructions, and a demonstration video (contact corresponding author to request material).

For the 3 week intervention period, all children with ASD completed nine computerized training sessions. These were 20–30 min in duration and occurred three times a week. The activities were all completed on a Dell Latitude laptop in the presence of the researcher. The children wore their RMHS while they were engaged in these training sessions. With agreement from each child with ASD, and the support of parents and teachers, the ASD group also wore their RMHS in school for the duration of the intervention period to maximize exposure to amplified hearing. Full details on the development of intervention and CAEP materials and processes are outlined in the supplementary material.

Session 3 took place the week immediately after the intervention period to evaluate intervention-related effects. The behavioral social perception assessment was re-administered, and CAEPs re-recorded. Finally, Session 4 occurred after a further two weeks. During this session, the behavioral social perception assessment was administered for the final time as an evaluation of the retention of any intervention-related effects.

The children from the TD control group only participated in a one-off session that included the hearing screen, parental report of communication behaviors, the behavioral social perception assessment, and recordings of their CAEPs. It is noteworthy that we were only able to test the TD children once each, rather than the four times for the ASD group, so the TD group cannot be considered a true control group. Rather, the TD group provided data that we used for cross-sectional comparison of behavioral and CAEP results.

We offered all participants the choice of having their behavioral assessments and training sessions (for the ASD group) at the University of Auckland’s clinic space, or in a quiet space in their own homes. It was important to ensure that the children with ASD had the opportunity to be in a space where they were comfortable and least anxious. We recorded all CAEPs at the University of Auckland in a sound-attenuating chamber.

### 2.4. Assessment Materials and Apparatus

#### 2.4.1. Hearing Screen

All participants received an evoked otoacoustic emissions screen measured via distortion product (DPOAE) using a Grason-Stadler GSI Audioscreener (version 3.21). OAE testing is a recommended hearing screening approach for young children [57,58]. The pass criterion was a signal-to-noise ratio (SNR) above 6 dB across five frequency levels (2–6 kHz) [59,60].

#### 2.4.2. Autism Characteristics and Communication Behaviors

The primary researcher, who met the requirement of qualification level C, observed the ASD participants during Session 1 and completed the high-functioning version of the Childhood Autism Rating Scale (CARS-HF) [61]. Results from the CARS-2 questionnaire for parents/caregivers (CARS-2-QPC) were used to complement and validate the autism symptom severity ratings given by the researcher. Standardized T-scores were derived from the raw total and corresponded to the following categories: between <20 and 41 indicates that individuals scored in the minimal–no symptoms of ASD range, between 42 and 50 indicates mild–moderate symptoms, and 51 and higher indicates severe symptoms of ASD.

Parents/caregivers of all children (TD and ASD) completed the second edition of the Childhood Communication Checklist (CCC-2) [62]. The CCC-2 screens for communication problems in children aged 4 to 16 years. Seventy questions make up 10 subscales that assess language structure, vocabulary, discourse, pragmatics (social rules of language), and impaired communicative behaviors commonly displayed by children with ASD. A General Communication Composite (GCC), scaled to individual age groups, indicated whether children may have clinically significant communication issues. Children with ASD (and specific language impairment) score below 55 according to validation data [62]. A Social Interaction Deviance Composite (SIDC), calculated separately, indicated whether an individual child may show a communicative profile that is characteristic of ASD. SIDC values below 0 are most commonly seen in children with Autism [62].

#### 2.4.3. Behavioral Social Perception

We assessed social perception using the Wechsler Advanced Clinical Solutions (ACS) Social Perception Subtests [63]. There were three subtests in total.

The ‘Affect Naming’ task required the participant to identify the emotion that is being expressed on a series of 24 facial photos. They were given a choice out of the following emotions: happy, angry, sad, afraid, surprised, disgusted, and neutral.

The ‘social perception face matching’ task required the participant to listen to an audio recording of a statement, and then select one facial photo out of six that they thought matched the emotional tone behind what was said. There was no need to verbally identify the emotion; participants were instructed to disregard the gender of the voice and the photos, and to focus on the affective facial expressions. There were cases where vocal-gender was incompatible with facial-gender. There were 12 items in this subtest.

The ‘social perception pair matching’ task followed a similar procedure as above, but instead of single faces, each item was accompanied by four photos depicting a scenario with two people. The participant chose one option out of the four, depending on which one they thought matched the emotional tone in the audio recording. Participants were asked to use the characters’ facial expressions and body language to inform their decision. Once again, some items out of the 12 had incompatible vocal- and facial-gender.

We derived two scores from these subtests—an ‘Affect Naming’ score, and a ‘Social Perception Prosody’ score (face- and pair-matching tasks combined), as per the ACS guidelines. Raw scores were used because published normative data are not available for individuals younger than age 16 years [63].

Average internal consistency across ACS scores is reported as *r* = 0.69–0.81, with test–retest reliability reported as a corrected coefficient of *r* = 0.60–0.70, and inter-rater agreement between 98 and 99% [63]. The Social Perception Subtests have previously been successfully administered to adults with high-functioning ASD, Asperger’s syndrome, and a typically developing control group [64], and revealed significantly worse performance from those with ASD compared to controls, only for social perception face- and prosody-matching tasks, but not Affect Naming.

#### 2.4.4. Cortical Auditory Evoked Potentials

##### Stimuli and Sequences

**Figure 1:**
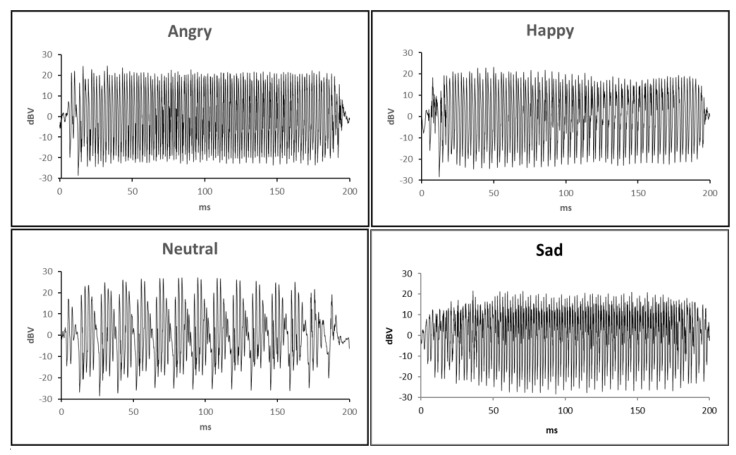
Electrical time waveforms (Adobe Audition CS6) depicting the monosyllabic stimulus/ba/stimulus presented in four emotions. dBV represents relative sound intensity in decibel-voltages, plotted against time in milliseconds (ms).

Speech stimuli were sampled from existing recordings of monosyllables (/ba/) produced by a male speaker in Angry, Happy, Sad, and Neutral emotional tones of voice [65]. See supplementary material for information on the selection of the four speech stimuli. All the speech stimuli were 200 milliseconds (ms) in length. The time waveforms in Figure 1 show that the speech stimuli were, on average, matched for root mean square intensity and duration but differed in their temporal characteristics.

We used a modified “oddball” paradigm to investigate auditory discrimination of Angry/Happy/Sad against Neutral. The stimuli were programmed into sequence blocks using the NeuroScan STIM2 Gentask software. Each block consisted of a ratio of 70 standard stimuli to 10:10:10 deviant stimuli. Every sequence began with 20 standard stimuli, followed by a pseudorandom presentation of standard and deviant stimuli. The order of emotional deviants inserted into the sequence was randomized but was adjusted so that at least 2 or 3 standards occurred between each deviant. There was a 640 ms inter-stimulus interval.

##### Experimental Setup and Data Acquisition

CAEP recording sessions took place in a sound-treated two-room setup, with a leather reclining chair for the participants to sit in. Stimuli sequences were presented (via Gentask software on NeuroScan STIM2) at 70 dB SPL via an Australian Monitor Synergy SY400 power amplifier and Sabine Graphi-Q GRQ-3102 equalizer, connected to a Turbosound IMPACT 50 loudspeaker. A half-inch polarized condenser free-field microphone, connected to a Bruel and Kjaer measuring amplifier and oscilloscope, was used to calibrate and externally monitor the sound levels of the stimuli in the enclosed testing environment.

We positioned the loudspeaker at a 150 cm distance at zero degrees azimuth in front of the participant seated on the recliner. Behind the loudspeaker was a television on a stand. We instructed participants to watch a movie of their choice with the audio turned off and the subtitles on, and to minimize their blinking and body movements during the recordings.

We recorded CAEPs using the NeuroScan Inc. Evoked Potential System (version 4.5) with a SynAmps 2 amplifier. Eight 10 mm gold electrodes were placed on Cz, Fz, F3, F4, A1, and A2 locations, with a ground electrode on the forehead, and an eye blink electrode above the right eye. The electrode on the right mastoid (A2) served as the reference electrode. During offline processing, we linked the left and right mastoid electrodes, and re-referenced Cz, Fz, F3, and F4. We kept electrode impedance at or below 5 kΩ and used a sampling rate of 500 Hz and a bandpass filter setting of 0.1–100 Hz.

##### Data Processing

Post-acquisition, we performed further offline processing using the Edit software from NeuroScan Inc. Continuous recording files were epoched from -100 ms pre-stimulus to 850 ms post-stimulus, followed by baseline correction. Any responses exceeding ±150 μV were rejected as artifacts. A minimum of 20 blinks were required to estimate an average blink. We filtered the data using a low-pass filter at 30 Hz (12 dB/octave slope, zero phase shift). We generated separate average files for each participant for each of the four emotions.

On average, approximately 20% of responses were rejected from the TD participants, as a result of ocular and other noise artefacts. For the children with ASD, an average of approximately 40% of responses were rejected.

All grand average waveforms are plotted from −100 ms to 850 ms to encompass pre-stimulus responses, the 200 ms stimulus length, and post-stimulus responses for the duration of the 640 ms ISI, without overlapping with the subsequent stimulus.

##### Data Analysis

The complexities of using emotions tones as auditory stimuli contribute to the current dearth of evidence in the mismatch response literature, and there is little consensus on what a “typical mismatch waveform” looks like and what components are expected to be observed. The heterogeneous nature of CAEPs recorded from clinical populations like children with ASD adds to this, thus we took some additional steps to maximize consistency in the analysis of individual waveforms and quantify latencies and magnitudes of specific waveform components in a consistent manner.

Firstly, we averaged responses across individuals within each group of waveforms (TD, Pre-intervention ASD, and Post-intervention ASD) to create grand averaged waveforms for the standard (Neutral) and deviant (collapsed across Angry, Happy, and Sad) stimuli. We then subtracted the standard waveform from the deviant waveform to create a difference waveform for each participant group, which was then collapsed across electrode sites.

We conducted single-sample *t*-tests on these difference waveforms at each millisecond. We highlighted contiguous time periods of more than 30 ms where the waveform deviated at a significance level of *p* ≤ 0.001 from 0 μV. These contiguous time periods formed “mismatch windows”.

We superimposed the mismatch windows back onto original CAEP recordings from each electrode site for each individual participant. They were used as references for peak latency and mismatch response (MMR) magnitude quantification. MMR magnitudes were calculated by taking an absolute average of the amplitudes included within ±20 ms either side of the peak latency for shorter windows (<100 ms), or by ±50 ms either side of an approximate midpoint for the longer windows (>100 ms).

Only the selected windows were used to provide comparable data between groups. Each individual participant had the following data for each relevant mismatch window, for each electrode site:

- Peak latency for Combined-emotions-minus-Neutral difference waveform,
- Peak latency for Angry-minus-Neutral difference waveform,
- Peak latency for Happy-minus-Neutral difference waveform,
- Peak latency for Sad-minus-Neutral difference waveform,
- MMR magnitude for Combined-emotions-minus-Neutral difference waveform,
- MMR magnitude for Angry-minus-Neutral difference waveform,
- MMR magnitude for Happy-minus-Neutral difference waveform, and
- MMR magnitude for Sad-minus-Neutral difference waveform.

#### 2.4.5. Statistical Analyses

The following statistical analyses (using IBM SPSS Statistics, v20.0) were conducted:

1. Related-samples analyses to determine whether behavioral results from the social perception assessment differed from each other at the two baseline time points, and at the two post-intervention time points.
2. Independent-samples analyses to compare behavioral social perception results between the TD and ASD groups (pre- and post-intervention vs. TD).
3. Related-samples analyses to explore the effects of the intervention on behavioral social perception within the ASD group.
4. Repeated-measures analyses of variance (ANOVAs) were conducted on peak latencies and MMR magnitudes within each mismatch window to explore differences between each electrode site (Cz, Fz, F4, and F3).
5. Independent-samples analyses to compare peak latencies and MMR magnitudes between the TD and ASD groups (pre- and post-intervention vs. TD), for Combined-emotions difference waveforms.
6. Related-samples analyses to explore the effects of the intervention on peak latencies and MMR magnitudes between the ASD pre- and post-intervention waveforms, for Combined-emotions difference waveforms.
7. Related-samples analyses to explore peak latencies and MMR magnitudes differences between emotions within each group of waveforms (TD, ASD pre-, and ASD post-intervention).

## 3. Results

### 3.1. Participant Characteristics

All participants passed the DPOAE screen, which indicated that they did not have significant middle-ear pathology or damage to the outer hair cells in the cochlea that would be associated with peripheral hearing loss. TD and ASD groups did not differ significantly from each other, and both groups were within normal range [66].

Autism characteristics from the CARS-2 ratings showed that nine of the 12 children in the ASD group obtained standardized T-scores in line with “minimal severity” (*M* = 33.44, *SD* = 3.21), and three of the children scored in the mild–moderate ASD severity category (*M* = 47.67, *SD* = 1.53). Results from the CARS-2 re-confirmed the diagnoses of all the children in the ASD group.

Table 1 details mean DPOAE signal-to-noise ratios (SNR), from left and right ears separately, measured in decibels (dB) across frequencies 2–6 kHz; group mean, minimum, and maximum T-scores from the CARS-2 (from ASD participants only); and group mean and standard deviations of General Communication Composite (GCC) and Social Interaction Deviance Composite (SIDC) scores from the CCC-2 (communication behaviors). Parental reports from the ASD group highlighted significantly more communication difficulties compared to the TD group, for both the GCC (*t*_(24)_ = −4.24, *p* < 0.001), and the SIDC (*t*_(24)_ = −2.55, *p* = 0.017).

### 3.2. Behavioral Social Perception

Behavioral results from the social perception assessment were all normally distributed, according to the Shapiro–Wilk test, with the exception of the Affect Naming scores at Session 3 and 4. Parametric paired-samples *t*-tests (and Wilcoxon signed ranks test for not normally distributed variables) were used to compare Affect Naming and Social Perception Prosody scores between Session 1 vs. 2, and Session 3 vs. 4. These scores (at baseline, and post-intervention) were not significantly different. Hence, results from Sessions 1 and 2 were averaged together to form a “pre-intervention” score; and Sessions 3 and 4 were averaged together to form a “post-intervention” score.

For the Affect Naming score, independent-samples *t*-test results showed that the TD group (*M* = 19.29, *SD* = 2.23) performed significantly better (*t*_(24)_ = −4.33, *p* < 0.001) than the ASD group pre-intervention (*M* = 15.37, *SD* = 2.37). Paired-samples *t*-test results showed that the ASD group improved their scores significantly after participating in the 3 week intervention period (*M* = 21.33, *SD* = 0.94) (*t*_(11)_ = −9.71, *p* < 0.001). Cohen’s *d* for repeated measures yielded an effect size of 2.96 for the pre- vs. post-intervention comparison. Independent-samples *t*-test results showed that the ASD group surpassed the TD group on Affect Naming performance post-intervention (*t*_(17_._99)_ = −3.12, *p* = 0.006).

**Figure 2:**
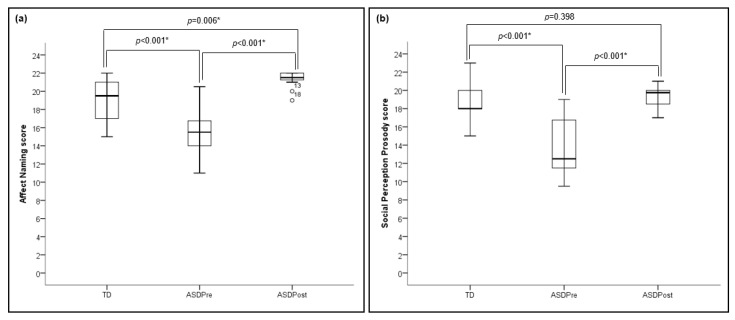
Behavioral social perception results from the Wechsler ACS Social Perception Subtests highlighting differences in performance between TD and ASD groups; and differences in performance pre- and post-intervention for the ASD group. (**a**) results for Affect Naming scores; and (**b**) results for Social Perception Prosody scores. * represent statistically significant differences.

For the Social Perception Prosody score, independent-samples *t*-test results showed that the TD group (*M* = 18.79, *SD* = 2.29) performed significantly better (*t*_(24)_ = −4.75, *p* < 0.001) than the ASD group pre-intervention (*M* = 13.75, *SD* = 3.10). Paired-samples t-test results showed that the ASD group improved their scores significantly after participating in the 3 week intervention period (*M* = 19.42, *SD* = 1.16) (*t*_(11)_ = −7.36, *p* < 0.001). Cohen’s *d* for repeated measures yielded an effect size of 2.05 for the pre- vs. post-intervention comparison. Independent-samples *t*-test results showed that the ASD group did not significantly differ from the TD group on Social Perception Prosody performance post-intervention (*t*_(24)_ = −0.86, *p* = 0.398). Figure 2 illustrates these results.

### 3.3. Cortical Auditory Evoked Potentials

**Figure 3:**
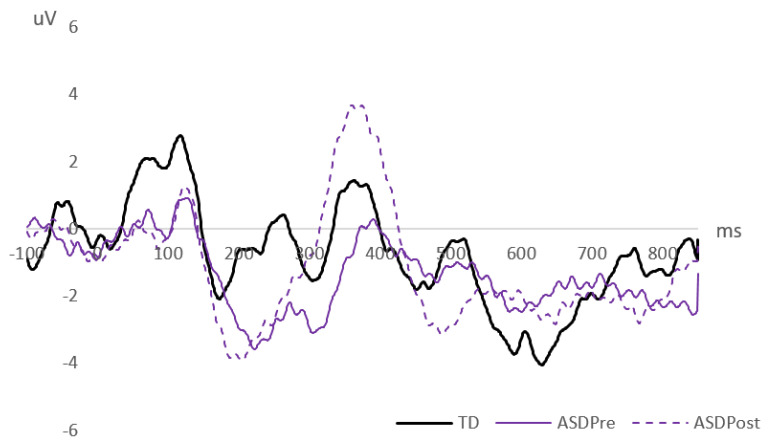
Difference waveforms (deviants-minus-standard), combined across emotions and electrode sites, resulting in a single grand average difference waveform for each group—TD children (thick solid [black] line), and ASD children pre- (thin solid [purple] line) and post-intervention (thin dotted [purple] line). Response amplitudes are measured in microvolts (μV) and time in milliseconds (ms).

**Figure 4:**
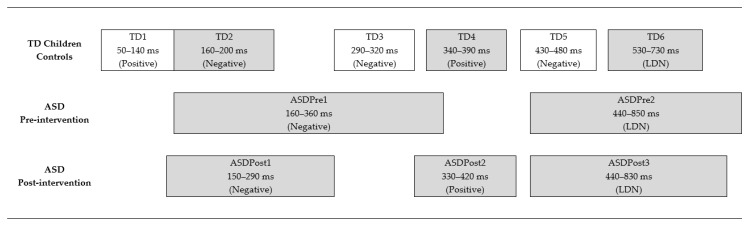
Timeline presentation of mismatch windows for each participant group, derived by conducting single *t*-tests at each time point of group average emotion-combined difference waveforms, and identifying contiguous periods of significant deviance from 0 μV.

Figure 3 shows the Combined-emotions difference waveforms derived for each group of waveforms (TD, ASD pre-intervention, and ASD post-intervention). As detailed in the Methods, single-sample *t*-tests were conducted on these difference waveforms at each millisecond. Contiguous time periods where the waveform deviated from 0 μV at a significance level of *p* ≤ 0.001 formed the “mismatch windows” illustrated in Figure 4.

Mismatch windows consisted of both positive and negative deviances, and ranged from 30 ms windows to longer periods, which were considered as late discriminative negativities (LDN). LDN components are thought to reflect the processing of more complex auditory stimuli, especially with regard to language and speech processing [67,68,69]. Long deviance periods were clearly identified in both groups of children in this study.

The shaded mismatch windows in Figure 4 represent the selected ones where peak latency and MMR magnitude data were computed. The first negative window (“*MMN*”) has data for all three groups (TD, ASD pre-intervention, and ASD post-intervention). The following positive window (“*MMP*”) has data for TD and ASD post-intervention comparisons. Data for the last negative window “*LDN*” can be compared across all three groups of waveforms.

Repeated-measures ANOVAs were conducted within each mismatch window to explore differences between electrode sites (Cz, Fz, F4, and F3). For the ASD group (pre- and post-intervention), there were no significant differences between electrode sites. For the TD group, there was a significant main effect of electrode for MMR magnitudes at two mismatch windows and peak latencies at one mismatch window. However, Bonferroni corrected post hoc comparisons revealed no significant differences between electrodes. Thus, peak latency and MMR magnitude data were averaged across electrode sites.

Table 2 details the results from the between-group (independent-samples *t*-tests) analyses comparing peak latencies and MMR magnitudes for Combined-emotions difference waveforms. Table 2 also details pre vs. post analyses (paired-samples *t*-tests) to explore the effect of the intervention on CAEPs for the children with ASD.

Figure 3 and Figure 4 show that at the first negative window—the MMN—the TD group demonstrated faster responses compared to the ASD group, when comparisons are conducted for both pre- and post-intervention waveforms. There were no significant differences in MMN amplitudes between groups. However, MMN latencies were significantly faster post-intervention for the children with ASD. At the positive window—the MMP—no statistically significant differences were found between responses from the TD group compared to the positive spike observed in the ASD waveform post-intervention. Finally, at the last negative window—the LDN—there were also no significant differences in the magnitude of responses.

Thus, the main group effect was for CAEP latencies; the ASD group pre-intervention showed significantly slower responses compared to the TD group. The significant increase in response speed post-intervention narrowed the gap, resulting in no significant latency differences between the TD and ASD groups after the ASD group received combined RMHS and computerized social perception training.

When the difference waveforms are examined for separate emotions, data were inconsistently normally distributed for both peak latency and MMR magnitude variables and hence non-parametric tests were used.

**Figure 5:**
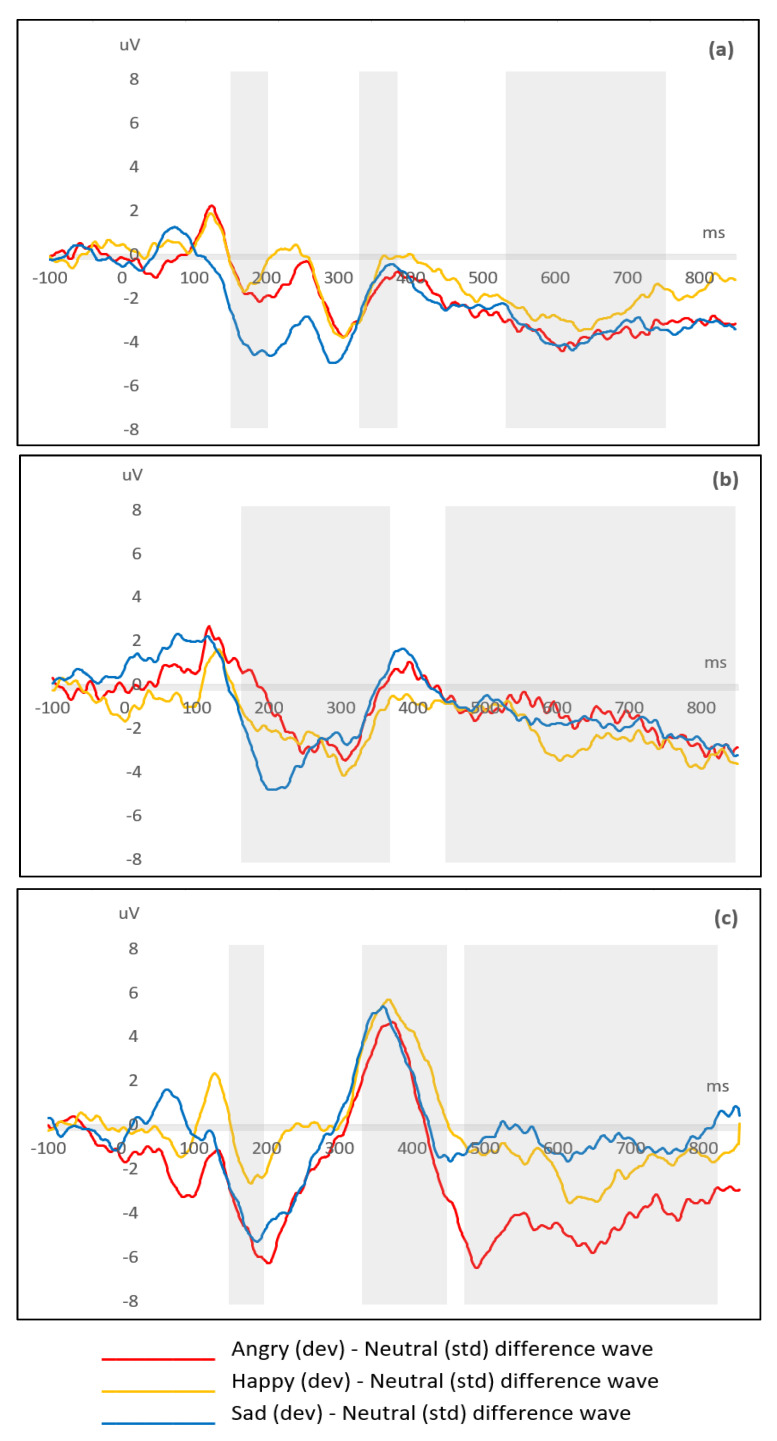
Separate emotion difference waveforms for (**a**) the TD group, (**b**) the ASD group pre-intervention, and (**c**) the ASD group post-intervention. Shaded regions depict the MMN, MMP, and LDN windows.

Figure 5 illustrates separate emotion difference waveforms for (a) the TD group, (b) the ASD group pre-intervention, and (c) the ASD group post-intervention. The shaded regions depict the MMN, MMP, and LDN windows where peak latency and MMR magnitude data were extracted and included in the related-samples analyses. Friedman tests revealed no significant latency differences between emotions for any group. Table 3 details Friedman analysis results for MMR magnitudes. For the TD group, there was a significant magnitude difference between emotions at the MMN window (*Χ*^2^_(2)_ = 7.02, *p* = 0.030). For the ASD group pre-intervention, there were no magnitude differences between emotions for any mismatch window. For the ASD group post-intervention, there were significant magnitude differences between emotions at the MMP (*Χ*^2^_(2)_ = 16.20, *p* < 0.001) and LDN (*Χ*^2^_(2)_ = 17.59, *p* < 0.001) windows.

## 4. Discussion

This study investigated how the perception of affective prosody (i.e., emotion) differs between children with ASD and their TD peers. It was hypothesized that difficulties with perceiving and understanding subtle nuances in speech may stem from underlying auditory processing challenges, thus a second aim of this study was to evaluate the effects of an auditory-based intervention with children with ASD. Affective prosodic perception was investigated via a behavioral social perception task and cortical auditory evoked response recordings.

### 4.1. Behavioral Results and Implications

Overall behavioral results showed that TD and ASD children (pre-intervention) did exhibit differences in their abilities to identify facial expressions (Affect Naming score), as well as matching facial to vocal emotions (Social Perception Prosody score). Future work could expand the collection of normative data from TD children so that raw scores from the ACS Social Perception [62] test can be standardized for ages younger than 16 years, the current cut off point for available standardized data. All participants were able to complete the ACS tasks successfully, indicating the suitability of the tool for younger ages.

Significant improvement in social perception abilities was seen in the children with ASD after receiving computer-based training accompanied by amplified hearing via RMHS during the 3 week intervention period. Abilities improved to the extent that the children with ASD surpassed the TD group (who received no intervention) on Affect Naming and matched the performance of their TD peers on Social Perception Prosody scores. The TD group were not tested twice to determine whether their scores would be stable over time, however, the test–retest reliability of these measures has been previously reported [63].

A number of studies report similarly successful computer-based training for emotion recognition [70,71,72,73] and attention to prosodic cues [74,75] in individuals with ASD. A recent review [76] revealed that the largest training-related improvements for individuals with ASD result from specifically targeting, for example, speech prosody, and interventions using evidence-based practices and spanning across more than one treatment session. A clear future progression from many of the existing studies is the integration of auditory with visual stimuli, as was done in this study, to improve social understanding and making the learnt skills more generalizable to the real world [45,77,78,79].

Studies that have investigated computer-based training alongside use of hearing amplification technology (RMHS) have largely focused on evaluating auditory processing specific outcomes measures, self-perceived hearing difficulties, and classroom listening behaviors [53,56]. The use of RMHSs in conjunction with prosodic-specific training has received little attention in the literature to date, and to our knowledge the effects on emotion recognition and social perception have not been reported previously.

### 4.2. Electrophysiological Results and Implications

This study demonstrated that the natural speech stimuli spoken with four different emotions, presented in an oddball paradigm, evoked measurable obligatory CAEP components and significant mismatch responses from children with and without ASD. Most previous auditory change-detection studies in the literature involve non-speech sounds and simple speech stimuli, which reliably elicit prominent mismatch negativities in children between 150 and 250 ms [80,81,82].

The presence of positive MMRs elicited by the TD children in this study is supported by other studies looking at the manipulation of speech-related factors [83,84]. Due to the complex nature of stimuli used in this study compared to earlier studies it was difficult to predict where mismatch would occur and hence the statistical identification of mismatch windows (Figure 3 and Figure 4) was useful for documenting the responses in TD and ASD children.

TD children displayed a more complex mismatch response compared to the responses of ASD children pre-intervention who, in contrast, displayed a simpler pattern that consisted of two large mismatch negativities (Figure 3 and Figure 4). The first negativity is consistent with the pattern reported in the literature for TD children for non-speech stimuli [80]. It is possible that the children with ASD processed the stimuli as simple sounds, as their mismatch response did not reflect the spectral and temporal complexity of the speech stimuli. Post intervention, the difference waveforms of the ASD children still showed mismatch negativity, but with significant reduction in latency, i.e., a more rapid neural response to affective prosodic changes (Table 2). This suggests that the combined intervention of computer-based training and a clearer speech signal received through the RMHSs was associated with improved auditory processing.

A large mismatch positivity, as observed in the TD group and the ASD group post-intervention, could be attributed to increased sensitivity towards changes in prosody. Consistent with this, other training studies involving TD children and different CAEP paradigms have also found evidence for improved post-training evoked responses in this latency region. For example, studies with normal hearing, neurotypical children have reported significantly enhanced P2 amplitudes after auditory discrimination training targeting different voice onset times [85,86], phoneme changes [87], and pitch [88]. A mismatch positivity was not present in the ASD group pre-intervention in the current study, but this emerged post-intervention, consistent with reports that the auditory evoked P2 is a potential biomarker of learning and plasticity [89].

In terms of individual emotion differentiation, TD children produced a significantly larger first mismatch negativity in response to the Sad emotion, whereas Angry and Happy response did not differ (Table 3). These findings suggest that emotion differentiation may primarily be pitch driven, as anger and happiness are characterized by increased mean pitch, pitch range, and vocal intensity, whereas these acoustic parameters are usually reduced for sadness, coupled with a slower rate of speech and longer inter-articulation silences [90,91]. Future work in this area would benefit from more sophisticated analyses of natural speech stimuli, which would better inform future studies regarding which parameters to manipulate. Delving deeper into whether emotion is primarily modulated by changes in pitch contours and temporal resolution, and its correlation with valence and arousal strength [92], may deepen our understanding of the emotion perception difficulties of individuals with ASD and other neurological conditions.

Children with ASD did not show any differences between emotions in their MMRs pre-intervention (Table 3). The grand average waveforms suggest that Sad is separable from Angry and Happy waveforms, but there are large variations between individuals and the sample size is small, which may account for the lack of significant difference. These results are consistent, however, with earlier behavioral [93] and neurophysiological [36,38] studies that show impaired emotion discrimination in children with ASD.

Post-intervention, the MMRs of the ASD group looked substantially different. As highlighted in Table 3, the children now show responses that differ between the three different emotions. These differences could have been driven by perceptual differences in vocal pitch and intensity between the three emotional stimuli. Another possibility is that this change does not reflect altered auditory processing at all. For example, it could be the result of pitch- and intensity-related heightened anxiety in individuals with ASD resulting in an altered attentional effect on the mismatch response. Studies of visual attention and facial discrimination report a dominant reaction towards threatening environmental stimuli [94]. Although changes in anxiety or other factors could have contributed to MMR changes over time, behavioral scores were stable prior to and after training, suggesting that MMR differences were more likely to reflect training effects.

Emotion differentiation occurred in the earliest MMN for TD children, suggesting pre-attentive neural activity that does not engage later-occurring higher cognitive processes. This was not the case for children with ASD. Significant emotion differentiation was evident at the MMN, the MMP, and the LDN time windows (i.e., throughout the mismatch waveform) post-intervention, which suggests both pre-attentive and conscious appraisal of emotional differences. Thus, the intervention may have enabled the children with ASD to differentiate the emotions, but did not ultimately ‘normalize’ underlying auditory processing.

### 4.3. Limitations and Future Directions

The effects of the intervention on the behavioral and CAEP data should be interpreted with caution, as one of the major limitations of this study remains that there were no alternative ASD groups who received different versions of the intervention, nor was there a control group of children with ASD that received no intervention. The method of administering the behavioral test of social perception four times (twice pre- and twice post-intervention), and the resulting lack of statistical differences between Sessions 1 vs. 2, and Sessions 3 vs. 4, strongly suggests that the changes in behavioral performance are attributable to the intervention and not to test–retest effects. However, the same cannot be said for changes in the CAEPs and should be addressed in future extensions of this work.

Data from the TD group had a number of limitations, due to the need to a) administer a full range of assessment measures (both behavioral and electrophysiological) within a tight time frame, b) minimize participant fatigue, and c) minimize the imposition on TD volunteers. These limitations included not evaluating the TD children with the CARS-2 to rule out autism symptoms, only testing them at one time point, and not providing the TD children with an intervention. Future studies with sufficient time and funding could improve on this by conducting a randomized controlled trial of various interventions with ASD and TD groups.

Hyper or hyposensitivity to sound was not specifically measured in either ASD or TD groups. This is a potential confounding variable, as demonstrated in other studies investigating the neural processing of auditory information [95]. All ASD and TD children tolerated the hearing screening using DPOAEs well, which involved presentation of tones at 55–65 dB SPL across a range of frequencies from 2 to 6 kHz. We cannot, however, comment on whether the children had atypical sound sensitivity or loudness perception. Future studies in this area could benefit from including a subjective measure of sound sensitivity (e.g., the Sensory Experiences Questionnaire [96], or measurements of loudness perception for the CAEP-evoking stimuli to determine whether these measures correlate with the mismatch response. There are also limitations to using DPOAEs as a measure of hearing, as it is most reliable for identifying the presence of moderate to severe hearing loss and does not offer a continuous measure of hearing thresholds [97]. Children with autism are at risk for peripheral hearing loss [98] as well as auditory processing difficulties [20], however with this clinical population, it is important to reduce the complexity of task demands to ensure that the quality of the data is upheld. Although desirable, use of the gold-standard method of pure tone audiometric testing may not be feasible for all children with autism and hence, in the current study, we relied on parent report and objective DPOAE measurement to screen for moderate or greater hearing loss. In future studies, it would be of interest to investigate the associations between sound sensitivity, hearing thresholds, and the neural processing of auditory information and vocal affect recognition [95,99]. This research could advance our understanding of electrophysiological biomarkers of autism, or quantifiable measurements of neural benefit from auditory-based interventions.

## 5. Conclusions

This study was motivated by evidence that individuals with ASD experience significant auditory processing difficulties [19,20,26]. It was hypothesized that this affects discrimination of prosodic cues such as stress, pitch, and emotion, which hinders understanding of affective speech [4,8,99]. Consistent with this, children with ASD had poorer social perception scores than TD children.

An intervention consisting of a combination of computer-based social perception training exercises, and a 3 week trial using RMHSs to provide an amplified hearing experience was administered to a group of children with ASD. Behavioral performance on measures of social perception significantly improved post-intervention. Electrophysiological results showed altered neural activity in response to changes in vocal emotion post-intervention in the ASD group. Improved performance and mismatch responses suggest that auditory training may improve the perception of affective cues in speech and that this may enhance social communication.

This study reports promising pilot data, but further work is required involving a larger sample size and a longitudinal study design. Control or sham conditions are also required to better understand the connection between underlying auditory processing difficulties, perceptual discrimination and neural processing of affective speech, and social perception and communication for individuals with ASD.

## Figures and Tables

**Table 1:** DPOAE (dB), CARS-2, and CCC-2 results from TD and ASD participants.

Measure		TD Group (*n* = 14)	ASD Group (*n* = 12)
DPOAE SNRs (right ear)	2 kHz	12.66	12.89
	3 kHz	14.62	14.63
	4 kHz	13.11	14.82
	5 kHz	13.69	14.37
	6 kHz	14.97	15.49
DPOAE SNRs (left ear)	2 kHz	12.97	14.64
	3 kHz	14.73	15.17
	4 kHz	13.35	15.77
	5 kHz	13.64	14.97
	6 kHz	15.75	15.40
CARS-2	Mean	-	37.00
	Minimum	-	29.00
	Maximum	-	49.00
CCC-2 (GCC)	Mean	66.36	38.42
	SD	18.92	13.75
CCC-2 (SIDC)	Mean	−1.86	−8.50
	SD	5.61	7.62

**Table 2:** Comparative analyses of peak latency and MMR magnitude data between three participant groups (TD, ASD pre-intervention, and ASD post-intervention) for combined emotions difference waveforms. * denotes group differences at a significance level of *p* ≤ 0.05.

Difference Waveform	Measure	Group 1 (M, SD)	Group 2 (M, SD)	Statistical Results
Combined Emotions—Neutral	Peak latency at MMN	TD (179.32, 10.11)	ASD Pre (259.10, 18.59)	*t*_(12_._81)_ = 12.33, *p* < 0.001 *
		TD (179.32, 10.11)	ASD Post (196.03, 9.01)	*t*_(22)_ = −4.17, *p* < 0.001 *
		ASD Pre (259.10, 18.59)	ASD Post (196.03, 9.01)	*t*_(9)_ = 8.42, *p* < 0.001 *
	Magnitude at MMN	TD (3.06, 1.60)	ASD Pre (5.05, 3.19)	*t*_(12_._27)_ = 1.81, *p* = 0.094
		TD (3.06, 1.60)	ASD Post (4.71, 2.96)	*t*_(22)_ = −1.77, *p* = 0.091
		ASD Pre (5.05, 3.19)	ASD Post (4.71, 2.96)	*t*_(9)_ = 0.34, *p* = 0.746
	Peak latency at MMP	TD (356.27, 45.85)	ASD Post (361.85, 11.43)	*t*_(15_._20)_ = −0.44, *p* = 0.668
	Magnitude at MMP	TD (4.46, 2.88)	ASD Post (5.27, 3.31)	*t*_(22)_ = −0.64, *p* = 0.532
	Peak latency at LDN	TD (618.20, 16.00)	ASD Pre (722.00, 38.76)	*t*_(11_._21)_ = 8.00, *p* < 0.001 *
		TD (618.20, 16.00)	ASD Post (633.70, 24.10)	*t*_(22)_ = −1.90, *p* = 0.071
		ASD Pre (722.00, 38.76)	ASD Post (633.70, 24.10)	*t*_(9)_ = 9.09, *p* < 0.001 *
	Magnitude at LDN	TD (5.32, 2.03)	ASD Pre (3.82, 2.89)	*t*_(22)_ = −1.50, *p* = 0.149
		TD (5.32, 2.03)	ASD Post (4.28, 3.64)	*t*_(22)_ = 0.90, *p* = 0.378
		ASD Pre (3.82, 2.89)	ASD Post (4.28, 3.64)	*t*_(9)_ = −0.52, *p* = 0.615

**Table 3:** Friedman analyses results (magnitude only) for all participant groups (TD, ASD pre-intervention, and ASD post-intervention) at each mismatch window. Additional information including median values, interquartile ranges, and individual Wilcoxon signed-ranks pair-wise comparison results are reported for significant main effects. * denotes emotion differences at a significance level of *p* ≤ 0.05.

Participant Group	Region	Friedman Results	Emotion 1 (*Mdn, IQR*)	Emotion 2 (*Mdn, IQR*)	Wilcoxon Results
TD	MMN	*Χ*^2^_(2)_ = 7.02, *p* = 0.030 *	Angry (2.50, 3.49)	Happy (2.89, 2.99)	*Z* = −0.52, *p* = 0.600
			Angry (2.50, 3.49)	Sad (5.00, 3.19)	*Z* = −2.67, *p* = 0.008 *
			Happy (2.89, 2.99)	Sad (5.00, 3.19)	*Z* = −2.73, *p* = 0.006 *
	MMP	*Χ*^2^_(2)_ = 1.71, *p* = 0.424	-	-	-
	LDN	*Χ*^2^_(2)_ = 0.11, *p* = 0.947	-	-	-
ASD Pre	MMN	*Χ*^2^_(2)_ = 1.40, *p* = 0.497	-	-	-
	LDN	*Χ*^2^_(2)_ = 5.00, *p* = 0.082	-	-	-
ASD Post	MMN	*Χ*^2^_(2)_ = 7.40, *p* = 0.025 *	Angry (7.21, 7.06)	Happy (4.14, 3.22)	*Z* = −2.50, *p* = 0.013 *
			Angry (7.21, 7.06)	Sad (8.06, 4.12)	*Z* = −0.15, *p* = 0.878
			Happy (4.14, 3.22)	Sad (8.06, 4.12)	*Z* = −2.09, *p* = 0.037 *
	MMP	*Χ*^2^_(2)_ = 16.20, *p* < 0.001 *	Angry (4.13, 2.32)	Happy (11.03, 4.31)	*Z* = −2.80, *p* = 0.005 *
			Angry (4.13, 2.32)	Sad (7.73, 2.96)	*Z* = −2.50, *p* = 0.013 *
			Happy (11.03, 4.31)	Sad (7.73, 2.96)	*Z* = −2.40, *p* = 0.017 *
	LDN	*Χ*^2^_(2)_ = 17.59, *p* < 0.001 *	Angry (9.44, 6.39)	Happy (4.74, 3.41)	*Z* = −2.67, *p* = 0.008 *
			Angry (9.44, 6.39)	Sad (3.96, 2.99)	*Z* = −2.80, *p* = 0.005 *
			Happy (4.74, 3.41)	Sad (3.96, 2.99)	*Z* = −2.09, *p* = 0.037 *

## Data Availability

Data (participants de-identified) supporting reported results in this study can be supplied on request by emailing the corresponding author.

## Supplementary Materials

A 3-part document is available online at https://www.mdpi.com/article/10.3390/brainsci11040469/s1. Part A includes details about remote-microphone hearing systems: product, verification, and familiarization period. Part B details the development of the computer-based social perception training. Part C describes the speech stimuli for cortical auditory evoked potential recordings.
