# Supplementary material for: Improving Emotion Perception in Children with Autism Spectrum Disorder with Computer-Based Training and Hearing Amplification
Source: Brain Sci. 2021 Apr 8;11(4):469. doi: 10.3390/brainsci11040469 (PMC8068114; doi:10.3390/brainsci11040469)
Supplement: Supplementary file 1 [file brainsci-11-00469-s001.pdf]

## SUPPLEMENTARY MATERIAL

### Part A: Remote-microphone hearing systems: product, verification, and familiarization period

*Remote-microphone hearing systems (RMHSs)*

RMHSs are a form of hearing technology that focuses on ameliorating hearing experiences in noise, by capturing speech signals close to the source (through a microphone on a transmitter) and wirelessly sending it to receivers worn by the recipient of the communicative exchange. This direct transmission aims to overcome background noise, distance, and poor room acoustics to deliver optimum audio information to the listener. For this study, hearing technology manufacturers – Phonak, provided six sets of their Roger wireless devices to trial with the sample of children with ASD.

Users of Roger transmitting technology paired with hearing aids [110, 111] as well as cochlear implants [112], have demonstrated significantly better performances on speech-in-noise sentence recognition tasks, especially when background noise was simultaneously presented at high levels (80dB), compared to users of older technology like dynamic or traditional frequency modulating (FM) transmitting devices. Study participants (blinded) who trialled the three types of RMHS technology in four different real-life noisy environments also reported that they preferred the Roger devices over dynamic and traditional FM systems [111].

According to the Phonak product reference guide, the Roger Inspiro transmitter has built into it an audio chip with proprietary programming that wirelessly sends signals at a 2.4 GHz (giga-Hertz) frequency band to connected receivers in approximately 17 milliseconds. It has a potential transmission range of 20 meters, compared to older FM technologies which can transmit over 30 meters in distance. The Roger system's more moderate range partially reduces the absorption of audio signals by obstructing animate and inanimate bodies. However, body absorption levels are still compromised in comparison to short range hearing aids, and therefore has limited appropriate situations where it could be optimally used. So far, the Roger Inspiro has been deemed most appropriate for classroom or lecture use, with the lapel microphone designed specifically for educational settings. Due to its fixed 2.4 GHz frequency band, users of the Roger system are also less likely to experience transmission interference from overlapping frequencies, and do not need to be concerned about constantly managing the frequency band of their own devices, which was a problem with the older FM technologies.

In addition, the Roger system has an in-built dynamic noise-adaptation function called EasyGain. Depending on immediate noise levels in the environment, the transmitter is capable of measuring the signal-to-noise ratio (SNR), and automatically adjusting how much signal gain is required to produce optimum audio information to be sent to the connected receivers. This feedback process repeatedly assesses the noise in the environment, and signal gain adjustments are constantly being updated. For this study, the Roger Inspiro transmitter was paired with two Roger Focus receivers, made specifically available by Phonak for individuals with auditory processing disorder (APD), ASD, and somatic symptom disorder (SSD).

*Verification procedure for RMHS fitting*

The verification procedure was provided by Phonak (New Zealand). These were developed based on the American Academy of Audiology hearing assistance technologies guidelines, which were further validated by Schafer and colleagues [113] with normal hearing children, recommending that

real-ear measures were conducted to ensure appropriate SNR gains were met. The same procedure was done in the same way for each ear.

Firstly, otoscopy was performed using a Riester e-scope (No. 2110-203) with disposable specula to check for any blockages in the ear canal. Once confirmed, a probe tube was inserted into the child's ear. A real ear unaided response (REUR) at a 65dB SPL stimulus presentation level was recorded on the AudioScan Verifit system. The corresponding Roger Focus receiver was then inserted into the ear, but remained turned off. A real ear aided response (REAR) at the same stimulus level was recorded, and checked to make sure the two electro-acoustic contours more or less overlapped with each other, to indicate minimal hearing disruption due to outer and middle ear obstructions.

The Roger Inspiro transmitter was then switched on and set to verification mode. The lapel microphone was aligned with the reference microphone inside a sealed testing box. The volume on the receiver was adjusted to maximum levels, and an electro-acoustic contour was recorded from a maximum power output (MPO) stimulus presentation. The child was asked if the sound level was too loud, and the volume on the receiver was adjusted accordingly until it was at a comfortable level. If the receiver volume had to be adjusted, another MPO stimulus was presented, and an updated electro-acoustic result was recorded. All participants had their transmitters set to EasyGain to avoid inter-user confound, and to make use of the automatic SNR gain adjustment technology that acclimatizes to varying classroom circumstances. Two speech sounds produced from the FM chest level were then presented, and checked to make sure both electro-acoustic contours did not exceed the MPO one. Key lock was activated on the transmitter and the receivers to prevent anyone from changing the settings while they handled the system.

***Familiarisation procedure for RMHS (adapted from Schafer et al., 2013)*** **[57]**

With permission from Dr. E. Schafer, from the University of North Texas, an adapted version of her "educational period" was implemented in this study. During this period, the children participants with ASD were given the opportunity to be familiarized with wearing and using the RMHS, and were briefed on what the intervention would entail. The familiarization period took up to one week to ensure that the participants were comfortable and confident with using the RMHS.

Day 1 involved the researcher visiting the participant at home, bringing along an AudioScan Verifit system, and one Roger Focus and Inspiro RMHS (2 receivers and 1 transmitter). The researcher measured the child's ear and attached appropriately sized tubing and ear insertion cones onto the two receivers. Following that, the participants underwent a verification procedure for their fitted RMHSs, which is detailed above.

Day 2 involved the researcher returning to the participant's home. The children were encouraged to read two 'social stories' together with the researcher – one about "wearing my Roger system", and the other about "how my Roger system is helping me". Lastly, the participants were shown a demonstration video made by the researcher. The production process of the video is detailed in a separate section below. After watching the video, the children proceeded to practice putting on the two receivers, initially with help from the researcher, and then by themselves under supervision. The researcher made sure that the child could successfully put them on and take them off unaided at least five times before the day was concluded.

For days 3-5 of the familiarization period, the participants were left with the RMHSs and spare batteries, and were encouraged to use them as much as possible at home before the start of the trial the following week.

*Production of the demonstration video to RMHSs*

With support from SoundSkills Auditory Processing Disorder Clinic (Auckland, New Zealand), one boy and two girls with APD, aged 10-12 years, who were users of the Phonak Roger system, were recruited to take part in the production of an introductory video that was shown to the study participants with ASD. Both the children and their primary caregivers consented to being filmed for this video, and for their first names to be used.

Building from Schafer's RMHS familiarization process [57], it was believed that having a video model of similar aged children using the hearing devices will help those with ASD acclimatize better to the RMHSs and the subsequent 3-week trial.

The video began with scripted segments on how to wear and operate the system, as well as an overview of how they should be trialled at school and at home while doing the compuer-based training exercises with the researcher. The video concluded with the Roger system users providing feedback about their experiences with the hearing devices and how it has helped them. The video was structured into individual sections that focused on conveying different information, with ample transition times between each one consisting of a section title and some music. The total video length was 8 ½ minutes.

The boy and one of the girls took part in the filming at the University of Auckland in a mock classroom set up. After introducing themselves and their Roger systems, one actor demonstrated how to put the receiver on, while the other explained the different parts of the system and how the transmitter and receivers operated. They then elaborated on how the system could be used in class and why it was beneficial. Followed by a reminder to take the receivers off before going out to play at lunch time, as well as bringing the whole system home every day after school.

The other girl was filmed in her home, with the scene set up to demonstrate what it will look like when the researcher visits participants at home to conduct the computer-based training exercises. After introducing herself, the actress once again went through how to wear the receivers and operate the system. The purpose of this repetition was to reassure the children with ASD, in case they became anxious about not being able to remember all the steps after they watched it the first time. The actress then elaborated on how the hearing devices would be used in conjunction with the nine training sessions. The microphone from the transmitter was clipped on as close as possible to the built in speakers of the laptop to enhance the salience of the speech samples from the tasks.

Following on from the instructional sections, the video proceeded to feature a compilation of comments from the three actors in response to the questions: 1) what was it like before you got your Roger system; 2) what has improved for you since you started wearing it; 3) is it hard or uncomfortable to wear; and 4) what do your friends think about your Roger system? Answers to the first question generally revolved around not being able to focus in class, understand teacher's instructions, and requiring multiple points of clarification from the teacher before being able to do the work. The three children all reported doing better academically and being more efficient at tasks after they started using RMHSs. One also commented on how it helped her "hear better when the air conditioning or the projector is on" in the classroom. Two of the children reported that they experienced a slight discomfort during the first few days of wearing the hearing devices. However, this was reduced significantly after a few days and they subsequently said that it "felt like a part of them" and "could not even feel it" was in their ear. Finally, all three children recounted positive experiences of wearing their RMHSs in the presence of their friends, which subsequently had the effect of reassuring the children with ASD that they should not be reluctant to wear the hearing devices in fear of being teased by their peers.

**Part B: Development of the computer-based social perception training**

Each of the nine training sessions were programmed by the researcher on Microsoft Excel. Each session had three parts to be completed:

1) "Which Emotion Is It?" - participants had to choose the correct facial expression out of three available options that matched a written emotion. There were 30 to complete for each session.
2) "Match the Emotion" – the first part required participants to listen to a recorded speech sample and choose the correct matching facial expression out of three. There were 12 to complete for each session. The second part required participants to listen to two recorded speech samples, and choose whether they matched or not in underlying emotion (forced-choice task). There were again 12 to complete for each session.
3) "Build an Emotion"- participants were presented with randomized facial parts (eyes, nose, and mouth), and were required to put the "puzzle" pieces together to build four facial expressions.

All the visual and auditory material were different for each session. The tasks were kept in the same order, and all the participants completed the nine sessions in sequence. Figure S1 illustrates what the computer-based training sessions looked like, and how the programming was organized. Coloured textboxes and images were assigned different macro instructions so that, when clicked on, they would lead to relevant sections of the program. Participants were given immediate feedback as to whether they answered correctly. This came as a large tick "Correct!", or a cartoon face with "Oops, no". There was no audio pairing to the feedback, because there was the risk of participants with ASD becoming fixated on a particular sound, which could, for example, lead to deliberate incorrect answers just to trigger the sound.

The material for the training sessions was gathered from a number of different sources. Images of faces with different emotional expressions were sampled from the Mindreading Emotions Library developed by Baron-Cohen and colleagues at the University of Cambridge, UK. The researcher purchased the CD version of the assessment tool that included various picture and video files. These were sorted into folders labelled with different emotions. Character portraits of both genders and from a wide range of ages were selected. Targeted emotions included angry, afraid, annoyed, disgusted, happy, neutral, sad, surprised, and worried. A randomly sampled selection of alternative facial expressions was used as the other available options in the forced-choice tasks.

Speech material for the "Match the Emotion" part was recorded from nine speakers of both genders (4 male, 5 female), across different ages (ranges from 14 – 65 years). The speakers were asked to say different sentences, sampled from the Bamford-Kowal-Bench (BKB) sentence lists, in six emotions – angry, afraid, happy, neutral, sad, and surprised. Sentences with the criteria that they had little/no emotional content were selected. For example; "children like strawberries" would not have been selected; whereas "the house had nine rooms" would. Each speaker was given a list of 30 sentences to say in the different emotions, which totalled 180 speech samples recorded for each speaker. Selected samples were included in "Match the Emotion" for all nine training sessions. Approximately equal samples were used from each speaker for each emotion. The objective was not not only have samples that most obviously conveyed an emotion, because this might result in only a few speakers' samples being selected. Rather, the aim was to expose the participants to a variety of ways different people would convey the same emotion.

These nine training sessions formed a novel set of tasks for the participants, who will not have encountered any of the material from the assessments, or previous computer and iPad games and applications. For copyright purposes, the sessions were solely used for the purpose of this research, and there are no plans to disseminate the training material publically.

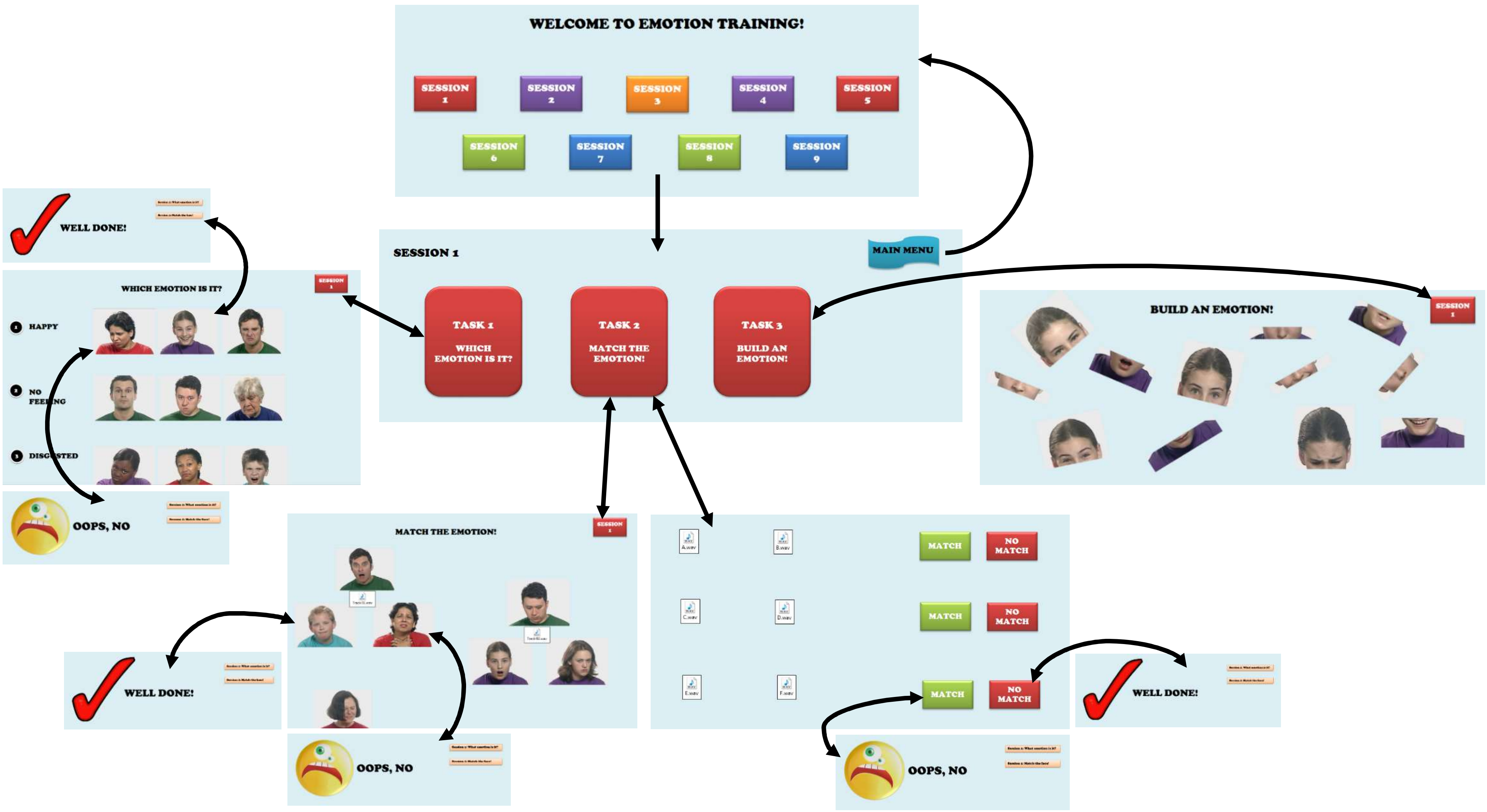


**Figure S1.** Illustration of how the programming of the computer-based training sessions were organized.

**Part C: Speech stimuli for cortical auditory evoked potential recordings**

Four speech stimuli – monosyllabic utterances /ba/ in angry, happy, sad, and neutral tones of voice – were used in an oddball paradigm to elicit cortical auditory evoked potentials (CAEPs) from the participants. These came from natural speech samples that were taken from a battery of prosody production tasks [74].

The original samples consisted of a string of seven /ba/ utterances, spoken by an actor in neutral, angry, happy, sad, surprised, and disinterested tones. The ones corresponding to angry, happy, sad, and neutral, were chosen, digitally cut, and edited using Adobe Audition software (version CS6) into individual monosyllables – each 200 ms in length, and on average 15 dBV in root mean square intensity. A total of 28 separate samples were derived from the originals.

Fourteen neuro-typical young adults with normal hearing were recruited to blindly rate the 28 monosyllables, with the task of identifying which of the four emotions they thought was being conveyed. The final four speech stimuli were selected, one for each emotion, because they received a 14/14 rater consensus and were all judged as conveying the intended emotion.

The CAEP recordings took place in a sound treated two-room environment. Prior to commencement of the study, measures were taken to adjust the intensity of the speech stimuli in relation to the testing environment. A sound level meter (Brüel & Kjaer, type 2215, Denmark) on an adjustable tripod was placed on a leather reclining chair at 50 cm in height, and 150 cm away from a Turbosound IMPACT 50 loudspeaker positioned centrally facing the chair. This recliner was where the participants sat during the recording session, and remained in a fixed position for the duration of the study. The stimuli was presented through the Gentask software on NeuroScan STIM2, and was incrementally adjusted until the sound pressure level (SPL, linear weighting) was 70 dB SPL +/- 0.9 dB. The loudspeaker remained in this fixed position for the duration of the study. Following a trial run, the researcher noticed some audible inter-stimulus interference, as there were some software to hardware delays between the stimulus presentation computer and the loudspeaker. 40 ms of silence (20 ms either end of the utterance) was added to each of the speech stimuli, which removed the noise, but retained the 200 ms of voiced time; this silence was accounted for in the post-processing of response.
